# Bibliometrically mapping the research field of entrepreneurial communication: where we stand and where we need to go

**DOI:** 10.1007/s11301-023-00355-3

**Published:** 2023-06-13

**Authors:** Manuel Kaiser, Andreas Kuckertz

**Affiliations:** 1 Entrepreneurship Research Group, University of Hohenheim, Wollgrasweg 49, 70599 Stuttgart, Germany; 2 Fraunhofer IAO, Fraunhofer Institute for Industrial Engineering IAO, Nobelstraße 12, 70569 Stuttgart, Germany

**Keywords:** Bibliometric analysis, Entrepreneurial communication, Historiography, Startup, Thematic map, L26, M13

## Abstract

Entrepreneurial communication is vital for acquiring resources and building stakeholder relations in startups. This research stream has grown rapidly in recent years and has developed as a multidisciplinary field at the interface of communication and entrepreneurship. However, this rapid development and the plethora of associated perspectives have led to a diverse and fragmented research field with different foci and concepts, making structural overviews difficult. Against this background, we conducted a bibliometric analysis to uncover the hidden structure of previous entrepreneurial communication research and to guide scholars toward a future research agenda. First, we identified 383 articles via the Scopus database, published in 245 academic sources, that covered nearly 50 years of research. We then connected the results of previous research using co-occurrence analysis and a thematic map to highlight the intellectual structure of the field and offer insights into its research clusters. Our algorithmic historiographic analysis illustrates the development of the field over time and highlights upcoming topics. Overall, entrepreneurial communication is crucial, particularly for startups engaging in resource acquisition for employee and investor relations with venture capitalists and business angels.

## Introduction

Communication is vital for entrepreneurs to overcome weaknesses and build relationships with their stakeholders and major resource providers (Fischer and Reuber 2014). Recently, research has seen an increasing number of studies addressing the communication activities of startups and entrepreneurs under the umbrella of entrepreneurial communication or startup communication (Fischer and Reuber 2014; Godulla and Men 2022; Gossel 2022; Wiesenberg et al. 2020). Broadly, we may equate entrepreneurial communication with all communication emanating from startups, but focusing on more group-oriented subcategories, such as investor relations (Kollmann and Kuckertz 2006; Moritz et al. 2015), public relations (Chen et al. 2021), or employee relations and leadership communication (Men et al. 2018, 2021a) makes entrepreneurial communication activities more concrete. We know from these previous studies that entrepreneurs must undertake multiple communication tasks to support their stakeholder relationships. Furthermore, since entrepreneurs rely heavily on these relationships (Pollack et al. 2017), from a strategic perspective, communication can play an essential role in ensuring an organization's survival (Zerfass et al. 2018).

As new actors on the market, entrepreneurs must signal that they are part of it, and in doing so, they must communicate to their different stakeholders their startups' existence (Sing and Aust 2022). Furthermore, to grow and survive, entrepreneurs need different resources from these stakeholders (Huang and Knight 2017). Moreover, this lack of resources is also countered by entrepreneurial communication as it is essential for resource acquisition (Martens et al. 2007; Wiesenberg et al. 2020); it helps to create legitimacy (Nagy et al. 2012) and is part of trust building (Kaiser and Berger 2021) with these stakeholders to create the exchange of resources. In this vein, previous research describes entrepreneurs' role as communication agents (Men et al. 2021a).

However, in today's volatile business environment, there is also a need for change and new requirements for entrepreneurial communication. On the one hand, the digital transformation, with its many different social media channels, is radically changing how entrepreneurs communicate (Olanrewaju et al. 2020), from a former personal level to online and sometimes anonymous mass communication. Moreover, this digital communication transformation process was accelerated by the COVID-19 pandemic (Statista 2020), which limited face-to-face communication (Ratten 2020). Thus, new opportunities associated with technological progress are influencing entrepreneurial communication, as external events (e.g., crises) are changing established behaviors. On the other hand, another transformation process—sustainability transformation (Hinderer et al. 2021; Hockerts and Wüstenhagen 2010; Johnson and Schaltegger 2020)—is underway, also shaping entrepreneurial communication. Furthermore, globalization is compounding these developments by connecting people in different cultural contexts, which is why entrepreneurial communication must consider cultural aspects (Godulla and Men 2022). Because of these developments, research and practice on entrepreneurial communication must deal with considerable complexity.

In an overview, Wiesenberg et al. (2020) provided the first assessment of the research status quo concerning entrepreneurial communication and identified six dimensions: *resource acquisition, internal communication*, *external communication*, *branding*, *entrepreneurs' communication,* and *strategic communication*. These different dimensions, each garnering many publications, produced a diverse field of research that is extremely complex and heterogeneous. In general, such a situation makes it difficult for researchers to take an overview, is challenging for further research development, and can hinder the expansion of knowledge (Kraus et al. 2021). Furthermore, entrepreneurial communication research is conducted at the intersection of communication research (Men et al. 2021a) and entrepreneurship research (Fischer and Reuber 2014), further complicating the situation. Consequently, in a recent editorial for a special issue on startup communication, Godulla and Men (2022) described the research stream in this field as scattered and called for a systematic unifying perspective.

Against this background, we conducted a bibliometric analysis to provide an overview of the current state of knowledge and the structure of entrepreneurial communication research (Block and Fisch 2020; Donthu et al. 2021; Zupic and Čater 2015). Hence, this study answers the research question regarding the thematic structures of entrepreneurial communication in published research and how the discourse has developed over time. Furthermore, the results allow us to propose how research on entrepreneurial communication could and should develop and which topics will be relevant for future research.

We examined a dataset of 383 articles by 849 authors, associated with 22,086 references, taken from the Scopus database to answer the research question. Such an extensive dataset does not lend itself to a structured literature review but makes bibliometric analysis preferable (Zupic and Čater 2015). Furthermore, given the broad scope of entrepreneurial communication research, bibliometric methods are potentially helpful in structuring the research field (Donthu et al. 2021).

A key finding of our thematic mapping was that previous research has focused on communication in the context of resource acquisition—employee relations for human resources and investor relations for financial resources. In particular, communication with investors is a vast field of research that has drawn significant attention, as indicated by the most influential articles in terms of citations.

With this paper, we make two contributions based on bibliometric analysis (Block and Fisch 2020; Donthu et al. 2021). First, as necessary in emerging and evolving fields (Moritz and Block 2022), we contribute to structuring the research on entrepreneurial communication, showing its thematic evolution. Due to the scattered research landscape, a reliable overview is missing so far (Godulla and Men 2022; Wiesenberg et al. 2020). Primarily through the interplay of two disciplines—communication and entrepreneurship—we contribute by revealing their content structures and identifying trends. Since Wiesenberg et al. (2020) have already highlighted initial research areas in their literature review, we extended their approach by further elaborating on the underlying structures of the entrepreneurial communication research field by using bibliometric analyses. Furthermore, our findings are summarized in an integrative framework. Thus, we are meeting the demand for further systematic perspectives in this field of research (Godulla and Men 2022; Wiesenberg et al. 2020). Second, building on our results, we suggest future research areas for entrepreneurial communication and propose a research agenda grounded in existing research.

We have structured the remainder of this paper as follows. In Sect. 2, we describe the methods and analytical techniques. The results concerning the descriptive structure of the research field follow in Sect. 3. The thematic analysis based on science mapping is then presented in Sect. 4. Building on these analyses and findings, we develop an integrative framework and suggest a research agenda for future entrepreneurial communication research in Sect. 5, give practical implications in Sect. 6, show the limitations in Sect. 7 and conclude the paper in Sect. 8.

## Methods

### Data collection

We used the Scopus database to identify relevant academic articles with an entrepreneurial communication focus which researchers employ widely for bibliometric analysis in entrepreneurship research (Anand et al. 2021; Dolhey 2019; Pellegrini et al. 2020). Previous research highlighted the enormous scope of the database (Anand et al. 2021) and the quality of the covered journals (Dolhey 2019). Scopus is a citation database that comprises over 84 million records, of which more than 26 million relate to peer-reviewed journals (Scopus 2022). Accordingly, this database is suitable for helping emerging research fields gain the broadest possible insight (Pellegrini et al. 2020); in our case into entrepreneurial communication developments (Godulla and Men 2022).

In selecting the keywords, we considered the diversity of entrepreneurial communication to obtain a comprehensive picture of this research stream. Thus, we considered entrepreneurial or startup communication (Godulla and Men 2022; Wiesenberg et al. 2020), impression management and self-presentation (Collewaert et al. 2021; Parhankangas and Ehrlich 2014), pitch presentations (Balachandra et al. 2021; Clingingsmith et al. 2022), investor relations (Moritz et al. 2015), public relations (Chen et al. 2021), storytelling (Chapple et al. 2021), rhetoric (Allison et al. 2013), and narrative (Martens et al. 2007; Williams et al. 2016). The following search terms emerged from these considerations: (entrepreneur* OR startup* OR “start-up*” OR “new venture*” OR “small firm*” OR founder OR SME OR “small enterpris*” OR “small enterpriz*”) AND (“impression management” OR communicat* OR pitch* OR “self-presentation” OR “self presentation” OR storytelling OR rhetoric* OR narrativ* OR “public relations” OR PR OR “investor relations”).

Overall, the first part of the search string covered different variations of *entrepreneurial,* and the second part covered the central concepts of *communication*, enabling us to search for entrepreneurial communication articles. This strategy also covered most of the keywords relating to Wiesenberg et al. (2020), extended in the communication part of the search string with more detailed keywords (e.g., impression management, pitch, and storytelling). However, our keywords differed from those of Wiesenberg et al. (2020) because we did not use marketing or branding keywords. Indeed, Wiesenberg et al. (2020) pointed out that their findings on startups' strategic communication covered two core areas: *entrepreneurial marketing* and *entrepreneurial communication*. Because our bibliometric analysis emphasized entrepreneurial communication, we focused on communication keywords. Nevertheless, we also identified entrepreneurial marketing articles but only those directly related to communication and thus part of *marketing communication* (Park and Loo 2022; Wallnöfer and Hacklin 2013).

Following previous bibliometric analyses, we used this search string to search the titles for relevant articles (Deyanova et al. 2022; Kalantari et al. 2017; Kraus et al. 2014) up to October 5, 2022.^1^ In line with these studies, the title search helped identify articles closely connected to our topic (Deyanova et al. 2022). Thus, we identified articles that addressed our research focus, and this procedure allowed us to access a larger dataset for entrepreneurial communication. The first search of Scopus returned 703 articles, but additional filters, which were used as exclusion criteria, reduced this finally to 383 articles.

**Fig. 1:**
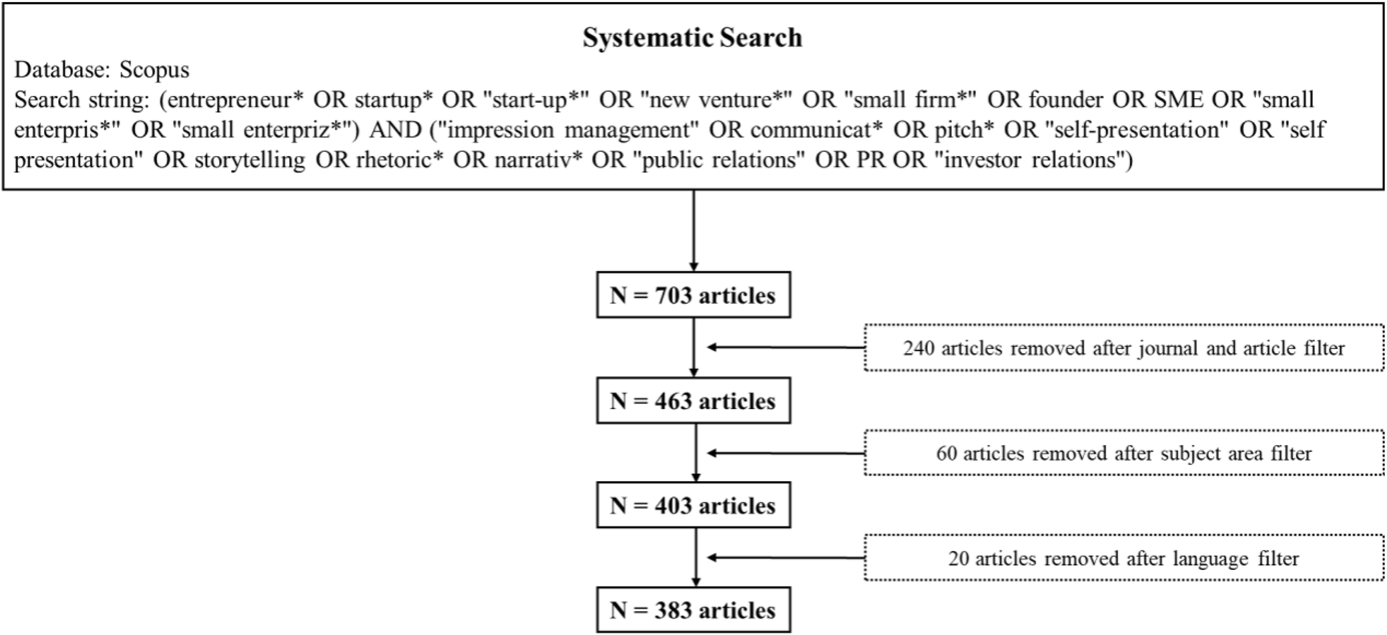
Article identification process

On the one hand, we included only journal articles for further analysis (Anand et al. 2021; Block et al. 2020). On the other hand, we limited the analysis to the Scopus categories *Business, Management and Accounting*, *Economics, Econometrics and Finance,* and *Social Sciences*. This approach enabled us to cover entrepreneurial communication articles in communication journals (Men et al. 2018, 2021a) and entrepreneurship or management journals (Davis et al. 2017; Martens et al. 2007) if they fell into different subject categories. For example, the *International Journal of Strategic Communication* is listed in Scopus under *Social Sciences* but has recently published articles on entrepreneurial communication (Godulla and Men 2022; Gossel 2022). Furthermore, we only included articles in the English language (Block et al. 2020; Deyanova et al. 2022). This procedure resulted in a final sample of 383 articles with 22,086 references. Figure 1 summarizes the data collection process.

### Bibliometric analysis

For various reasons, this study relied on bibliometric analysis to uncover the structure of previous entrepreneurial communication research (Block and Fisch 2020; Donthu et al. 2021; Zupic and Čater 2015). First, bibliometrics are helpful for broad areas of research (Block and Fisch 2020; Donthu et al. 2021), which was the case with the present study covering 383 articles. In addition, this research area contains various subcategories of communication (e.g., employee relations, investor relations, and public relations). Second, although earlier researchers worked only with print journals, there has been a rapid increase in digital publications, posing the challenge of managing large volumes of publications (Kraus et al. 2021). Therefore, bibliometric analysis methods allow for analyzing massive amounts of data without cognitive limitations (Pellegrini et al. 2020). Such an analytical approach can reveal research structures based on quantitative methods (Zupic and Čater 2015). Third, the main objective of our study was to summarize entrepreneurial communication research, identify its overarching structure (Block and Fisch 2020), and explore emerging patterns (Donthu et al. 2021).

Because various techniques are available for bibliometric analyses (Donthu et al. 2021), we briefly explain the techniques we employed to achieve our research goal. In detail, we used the Bibliometrix R package with the Biblioshiny application for the central part of our analysis (Aria and Cuccurullo 2017)—valuable tools used for previous bibliometric analysis (Forliano et al. 2021; Singh and Walia 2022). We also used CitNetExplorer (another relevant tool for conducting bibliometric analysis) to complement this approach (van Eck and Waltman 2014).

To gain an initial overview of the themes in entrepreneurial communication research, we used a *co-occurrence* analysis and employed relevant keywords. Co-occurrence (sometimes co-word) analysis provides an overview of the structure of a research field by analyzing the relationships between words (Zupic and Čater 2015).

For the visual preparation of co-occurrences and detailed analysis, we used a *thematic map* (Cobo et al. 2011) to cluster the research themes into four fields to assess their initial relevance (Aria et al. 2022; Aria and Cuccurullo 2017). To illustrate the evolution of entrepreneurial communication research over time, in addition to purely descriptive analysis, we also prepared a *historiograph* (Garfield 2004). Historiography shows how prominent individual articles are related to others across a timeline. These two analyses formed the core of our study of entrepreneurial communication research. We supplemented the content analysis with further descriptive analyses by examining our research field's developments over time and essential journals in the field. In summary, we incorporated both performance analysis and science mapping into our study. Table 1 summarizes our main techniques with short descriptions and the key tools utilized.

**Table 1:** Bibliometric analysis techniques

Main analysis	Description	Key tool for data analysis
*Descriptive analysis*		
Publications per year	Analysis of publications over time and development of the research field based on the number of publications	Bibliometrix
Top contributing journals	Analysis of the most influential journals based on the number of publications and publications per journal over time, with their journal rankings	Bibliometrix
Top contributing articles	Analysis of the 10 most influential articles (based on citations) and their contributions to the research field	Bibliometrix
*Science mapping*		
Thematic mapping	Analysis of the thematic clusters based on the keywords and their co-occurrences	Bibliometrix
Historiographic mapping	Analysis of the most influential articles based on a citation network and presentation over time	CitNetExplorer

## Descriptive map of the field

### Evolution of entrepreneurial communication as a research field

The first article identified in this analysis was published in 1973, so this bibliometric analysis covers the period 1973–2022 (up to October 5, 2022). However, all articles before 2003 were bundled together for a better overview. Before this period, research on entrepreneurial communication was relatively sparse and produced only a few articles per year (i.e., 21 articles were published from 1973 to 2003). The most productive year for publications was 2022, with 52 articles identified during the data collection in October, followed by 2020 and 2021, each with 49 articles. Overall, the results showed that 201 of the 383 articles were published between 2018 and 2022, meaning that 52% of the sample fell into this period. Research on entrepreneurial communication gained momentum during this period and developed from an emergent phase to a growth phase.

**Fig. 2:**
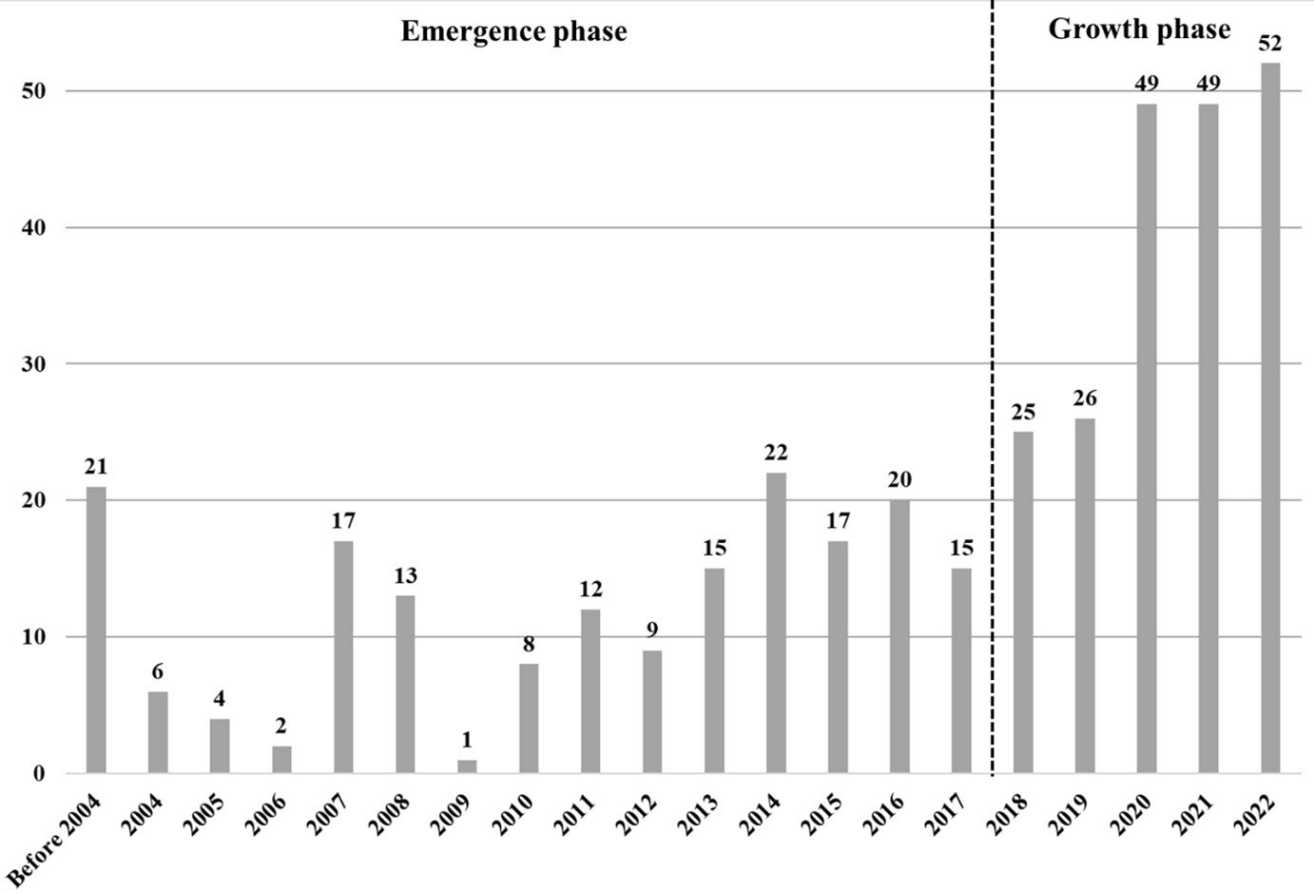
Articles per year in Scopus

The first 9 months of 2022 included more than twice as many publications as the first period (1973–2003). There may be many reasons for this. First, 2020–2022 was a particularly productive period, during which the COVID-19 pandemic struck, and researchers might have used the lockdowns to write articles (n = 49 in 2021 and 2020; n = 52 in 2022). In our case, this meant more research on entrepreneurial communication. Second, the data revealed that “new” communication researchers have recently become increasingly involved in entrepreneurial communication studies and have influenced the number of publications. This situation suggests that other disciplines have boosted entrepreneurial communication. Figure 2 shows the development of entrepreneurial communication research based on published articles per year, naming the different developmental phases.

### Important journals for entrepreneurial communication research

The 383 articles in this sample were published in 245 journals. The most productive journal was the *Journal of Business Venturing,* with 16 articles, followed by the *International Journal of Strategic Communication,* with 12 articles. This constellation further highlighted that entrepreneurial communication lies at the intersection of communication and entrepreneurship since the two most productive journals each covered one of these academic disciplines. The *Journal of Business Venturing* was the most influential in terms of total citations (TCs), with 1421 citations for 16 articles to date. However, the *International Journal of Strategic Communication* was the journal with the second least citations (TCs: 8) among our top 15 rankings due to the recency of the articles (11 of the 12 articles in this journal were not published until 2022). In the following places were *Entrepreneurship: Theory and Practice,* with eight articles, and *Sustainability,* with eight articles. Regarding citations, the *Academy of Management Journal* occupied second place (TCs: 817) with four overall articles, followed by *Entrepreneurship: Theory and Practice* in third place (TCs: 542).

Table 2 lists the top 15 journals according to the number of publications, supplemented by further information, such as the journal ranking according to different rating scores. The table includes 15 journals publishing at least four articles. Overall, the fragmented structure of the research field was reflected in the distribution of the 383 articles across the journals. The 15 most productive journals published 95 of the 383 articles, corresponding to nearly 25%, whereas 230 journals published only 1–3 articles.

**Table 2:** Top contributing journals in Scopus

Rank	Journal	No of articles	Before 2004	2004–2008	2009–2013	2014–2018	2019–2022	Total citations	SJR	ABS
1	Journal of Business Venturing	16	–	2	4	4	6	1421	5.829	4
2	International Journal of Strategic Communication	12	–	–	–	1	11	8	1.389	–
3	Entrepreneurship Theory and Practice	8	–	–	2	4	2	542	3.353	4
4	Sustainability Switzerland	8	–	–	–	2	6	93	0.664	–
5	IEEE Transactions on Professional Communication	6	–	–	–	5	1	74	0.445	–
6	Journal of Business Research	6	1	1	–	–	4	340	2.316	3
7	International Journal of Entrepreneurship and Small Business	5	–	3	–	1	1	23	0.287	2
8	International Small Business Journal	5	–	1	4	–	–	513	1.819	3
9	Journal of Business and Technical Communication	5	–	–	1	2	2	49	0.911	–
10	Academy of Entrepreneurship Journal	4	–	–	–	1	3	7	–	–
11	Academy of Management Journal	4	1	1	–	–	2	817	10.874	4*
12	International Journal of Entrepreneurial Behaviour and Research	4	–	–	1	1	2	43	1.206	3
13	Journal of International Entrepreneurship	4	–	1	–	1	2	76	1.004	1
14	Small Enterprise Research	4	–	2	–	–	2	11	–	1
15	Technological Forecasting and Social Change	4	–	–	–	1	3	49	2.336	3
	∑	95	2	11	12	23	47	4066	–	–

Furthermore, we used two established rating scores to evaluate journal quality: the SCImago Journal Rank (SJR) and the Association of Business Schools Ranking (ABS). The journal with the highest SJR (10.874) and ABS values (4*) in this sample was the *Academy of Management Journal*, with 4 articles out of the overall 383 articles. Other top-rated journals included the *Journal of Business Venturing* (SJR, 5.829; ABS, 4), *Entrepreneurship: Theory and Practice* (SJR, 3.353; ABS, 4), *Journal of Business Research* (SJR, 2.316; ABS, 3), and *Technological Forecasting and Social Change* (SJR, 2.336; ABS, 3). This result indicated that the journals with the highest (SJR and/or ABS) rankings mainly originated in the management or entrepreneurship literature but that entrepreneurial communication research from a communication perspective is growing.

### Important articles in entrepreneurial communication research

Although older articles often included a higher number of citations, this overview is heterogeneous, so the articles with the most frequent Scopus citations were published in the period 1996–2017. The article with the most frequent citations (TC: 466) was published by Martens et al. (2007). In their paper, the authors discussed the use of narratives, especially storytelling, in the context of resource acquisitions and showed how they influenced decision-making processes. In second place for the most frequent citations (TC: 275) was Sapienza and Korsgaard (1996), with their study on communication through feedback in the entrepreneur–investor relationship showing that feedback supports the positive shaping of investor relations. Third-ranked for citations (TC: 265) was Davis et al. (2017), with a further article on communication to acquire financial resources. In the context of entrepreneurial crowdfunding activities, the authors examined communication in online pitches and showed that communication product creativity could positively influence the acquisition process. The following article in this ranking (TC: 232) was the Introduction to the special issue *Entrepreneurial Narrative: Greif Symposium on Emerging Organizations,* written by Gartner (2007). This article provided an initial overview of the use of narratives for entrepreneurship research. Next was Rae (2005), with an article on entrepreneurial learning (TC: 205). This article referred to communication in a broad sense, analyzing the life stories of startup founders. The results of this study build a triadic model of entrepreneurial learning, meaning that entrepreneurial communication was a central part of the research design. Garud et al. (2014b) and their article on entrepreneurial storytelling followed in sixth place (TC: 204), examining projective storytelling to take a closer look at its effects on generating legitimacy. In their theoretical paper, the authors highlighted various possibilities and pointed to challenges in implementation. In seventh place (TC: 199) was Garud et al. (2014a), who examined how entrepreneurs use narratives in their communication to contextualize their innovations. The next (TC: 169) was Padilla and Pagano (1997), with their research on communication in the financial context of banks. Another article on communication for resource acquisition, by Allison et al. (2013) and ranked ninth (TC: 166), was devoted to rhetoric, examining how it affects microlending and showing that the communication context influences decision speed. The results suggested that communicated innovativeness is associated with greater investor risk and can influence the investment pace. Finally, the oldest paper in this ranking was Gassenheimer et al. (1996), ranked tenth (TC: 160). This article dealt with communication in the context of entrepreneurial cooperation, especially in the context of franchise systems. As a key result, the authors showed that communication can influence satisfaction in cooperative relationships.

Based on this initial analysis of the leading articles (according to citations) within entrepreneurial communication research, we noted that communication with resource providers, particularly investors, has been a leading research stream. Investor communication is, therefore, a defining area of previous entrepreneurial communication research. It is also interesting that the authors of previous research approached this communication from different directions, such as *narrative*, *rhetoric,* or even *storytelling*. Moreover, the *Journal of Business Venturing* was again in the lead for the number of articles and its influence on the ranking. Thus, these results showed that 3 of the 10 seminal papers were published therein. Table 3 provides an overview of the top 10 articles sorted by Scopus citations.

**Table 3:** Top 10 contributing articles per citation in Scopus

Rank	References	Title and journal	Total citations	Key results
1	Martens et al. (2007)	Do the stories they tell get them the money they need? The role of entrepreneurial narratives in resource acquisition. *Academy of Management Journal*	466	Narratives in entrepreneurs' communication support the acquisition of external financial capital
2	Sapienza and Korsgaard (1996)	Procedural justice in entrepreneur-investor relations. *Academy of Management Journal*	275	Communication through feedback supports the positive shaping of entrepreneur-investor relationships
3	Davis et al. (2017)	Funders' positive affective reactions to entrepreneurs' crowdfunding pitches: The influence of perceived product creativity and entrepreneurial passion. *Journal of Business Venturing*	265	Showing creativity in an online pitch of crowdfunding campaigns influences the funding performance
4	Gartner (2007)	Entrepreneurial narrative and a science of the Imagination. *Journal of Business Venturing*	232	n/a (Editorial to a special issue)
5	Rae (2005)	Entrepreneurial learning: a narrative-based conceptual model. *Journal of Small Business and Enterprise Development*	205	Narrative as a research method to build a conceptual model of entrepreneurial learning
6	Garud et al. (2014b)	Entrepreneurial storytelling, future expectations, and the paradox of legitimacy. *Organization Science*	204	Gaining legitimacy is linked to the recipient's expectations (cognitive and pragmatic)
7	Garud et al. (2014a)	Contextualizing entrepreneurial innovation: a narrative perspective. *Research Policy*	199	Narratives are a method used by entrepreneurs to market their innovations
8	Padilla and Pagano (1997)	Endogenous communication among lenders and entrepreneurial incentives. *Review of Financial Studies*	169	When banks share information about their customers, it can reduce and increase the bank's profit
9	Allison et al. (2013)	The effect of entrepreneurial rhetoric on microlending investment: an examination of the warm-glow effect. *Journal of Business Venturing*	166	The type of narratives influences the speed of funding
10	Gassenheimer et al. (1996)	Cooperative arrangements among entrepreneurs: An analysis of opportunism and communication in franchise structures. International* Journal of Business Research*	160	Satisfaction and performance are closely linked to communication

## Science mapping

### Conceptual structure with thematic mapping

To show the conceptual structure of entrepreneurial communication research, we built a thematic map using Bibliometrix (Aria et al. 2022; Aria and Cuccurullo 2017). Based on the co-occurrence of the authors' keywords, we identified the first thematic clusters within the research area (Block et al. 2020).^2^ The thematic map then helped us concretize the identified networks and, in particular, compare them in a matrix to obtain a detailed analysis of the co-occurrences (Aria et al. 2022). This procedure made it possible to evaluate research topics in four clusters: *niche*, *motor*, *emerging/declining,* and *basic* (Cobo et al. 2011). These four clusters are now briefly described based on explanations provided by previous studies (Aria et al. 2022; Cobo et al. 2011; Forliano et al. 2021).

*Niche themes* are specialized topics with minor relevance to the research area but have connections to other low-relevance topics. In contrast, some topics were highly important to the research field and well developed—the *motor themes*. The basic themes were less developed but equally important. Finally, emerging or declining topics lack development and are likewise of relatively marginal importance, so we summarized them under *emerging/declining themes*. These four clusters fell along two axes: the X-axis, which described the relevance of a topic (relevance degree), and the Y-axis, which indicated the stage of development (development degree).

*Motor themes* We included three directly assignable clusters in our thematic map. The largest cluster in our sample was highly developed and the most relevant, represented by the terms communication, the abbreviation for small-medium sized enterprises (SMEs), and innovation. Another cluster included entrepreneurial learning, resilience, and entrepreneurial storytelling. The third motor theme bundled impression management, business angels, and communication skills. Two other clusters were identified during the transition to basic themes. One cluster included startup, social media, and leadership; the other represented entrepreneurship education, entrepreneurialism, and higher education.

*Basic themes* We identified four clusters of basic themes. The first cluster, with the highest relevance but the least development, included entrepreneurship, narrative, and crowdfunding. Content analysis, narrative analysis, and entrepreneurial narrative constituted a further (second) cluster with a lower degree of development. The remaining themes fell into the third cluster, including legitimacy, marketing communication, and international new ventures, and the final cluster (Cluster 4), related to the keywords human capital and narrative paradigm.

*Emerging/declining themes* We bundled the topics in this map into five direct clusters with approximately similar values for relevance and degree of development. Four of these clusters had in common that they contained only one keyword: Cluster 1 referred to communicative competence, and Cluster 2 to competences. Cluster 3 referred to entrepreneurial networks, Cluster 4 to entrepreneurial passion, and Cluster 5 to performativity and process.

*Niche themes* Overall, we identified seven small clusters in this quadrant. Similar to the emerging/declining themes, we found three clusters with similar values for their relevance and development degree. Cluster 1 included the keywords categories and cultural entrepreneurship and was the cluster with the highest degree of development but a low value for relevance. Next was Cluster 2, including investment, pitching, and experiment, followed by Cluster 3, including agency and education policy, and Cluster 4, including intersectionality and organizational identity. Cluster 5 included collaboration, communication strategy, and open innovation; Cluster 6 included evaluation and the abbreviation for fuzzy-set qualitative comparative analysis (fscqa); and Cluster 7 included India, narrative inquiry, and narrative policy framework.

**Fig. 3:**
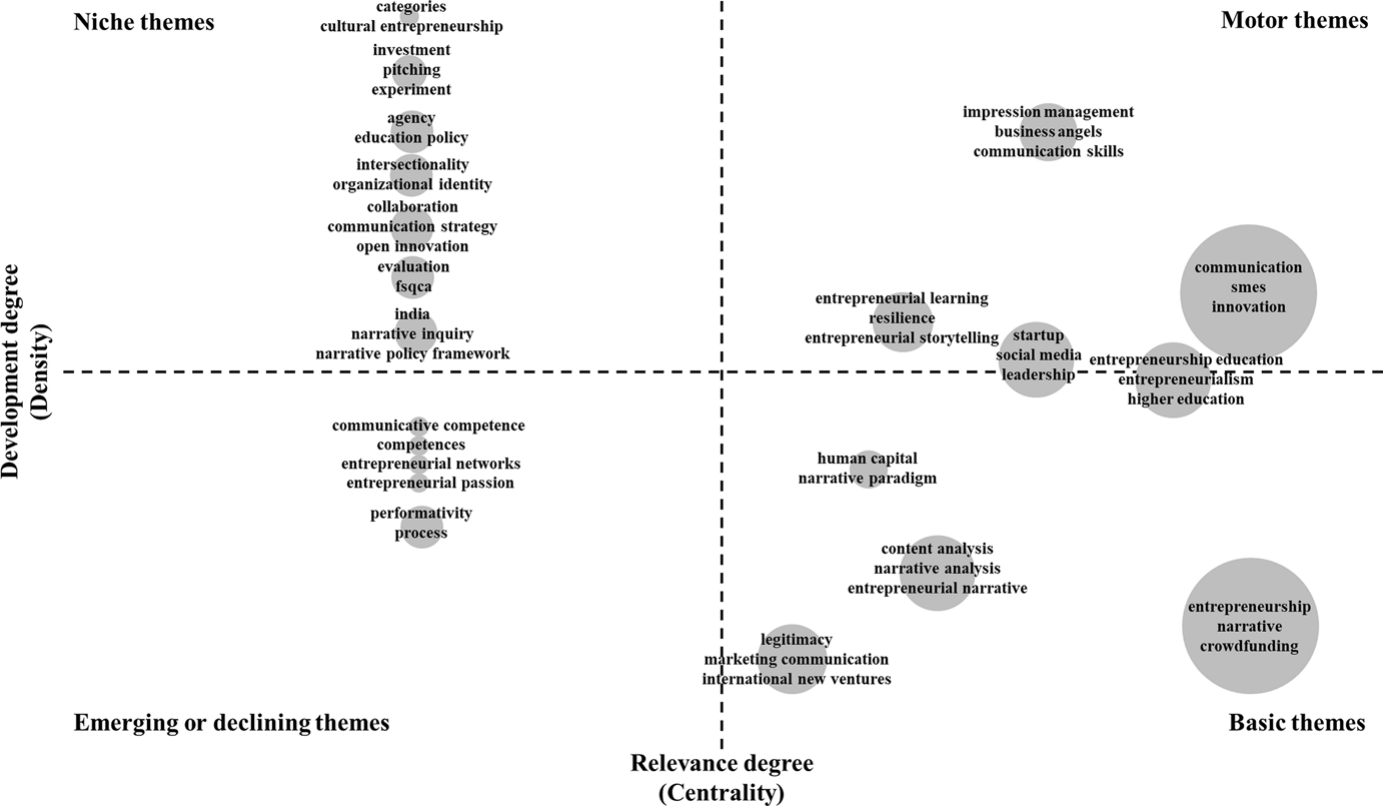
Thematic map of entrepreneurial communication research

 To ensure the readability of the figure, we restricted the clusters to a maximum of three words. In addition, we adjusted the circles in *Emerging/declining themes* and *Niche themes* to make the clusters more readable since, initially, these clusters lay directly on top of each other in their quadrants and were not readable. Based on the summarizing table and the visualized clusters in the thematic map, we noticed that impression management and business angels emerged as important motor themes for entrepreneurial communication in the upper right quadrant of the matrix. Overall, the relevance of stakeholder communication was evident in different clusters. Figure 3 shows the four quadrants with their thematic foci.

### Intellectual structure with historiographic mapping

Having already gained an initial insight into the development of the research field through the descriptive analysis (Sect. 3.1), we now sought to deepen the analysis. To understand the development of entrepreneurial communication research and its intellectual structure in a temporal context, we used a historiograph. This approach made it possible to understand core papers in their temporal contexts and their relationships to each other. Historiographs provide insights into the citation network of a research field and how documents are connected (Garfield 2004; van Eck and Waltman 2014). The result of such an analysis is a timeline that shows the years of publication for core publications and uses lines to visualize the citation relationships between documents. This approach provides an overview of how individual studies have contributed to developing a research field over time (Garfield 2004) and helps uncover influential studies in a chosen research field (Budler et al. 2021). We developed an algorithm-based historiograph using CitNetExplorer, following van Eck and Waltman (2014). Since this tool is primarily used for Web of Science datasets, we used the R package Scopus2CitNet (RStudio 2023) to prepare data for entry into CitNetExplorer and facilitate processing the Scopus dataset. We then analyzed and visualized our dataset using CitNetExplorer, using a minimum of 10 citations.

As mentioned above, historiographic mapping is an algorithmic analysis of a research stream and presents its core documents as a citation network (Garfield 2004). It is, therefore, a method to aggregate the topics of a research field (Kuckertz and Block 2021). In this vein, the algorithm analyzes the connection between articles through their citations, identifies core documents, and shows how topic clusters evolve (Vogel et al. 2021). The historiography results are visualized in a map showing the chronological development of the research field over time. In doing so, the Y-axis represents the timeline with the years the main articles are published (Budler et al. 2021). While traditional citation analysis primarily identifies clusters, the historiographic map extends this with the connection of the publication year and presents a timeline. Overall, this algorithmic-driven form of analysis and visualization of core documents and networks is emerging in the management and entrepreneurship literature. For example, Budler et al. (2021) use this approach to give an overview of the business model research and its underlying network, Bretas and Alon (2021) apply it in the context of franchise research, Ghura et al. (2022) provide a picture for corporate entrepreneurship and Alnajem et al. (2021) use historiographic mapping for the circular economy research stream.

**Fig. 4:**
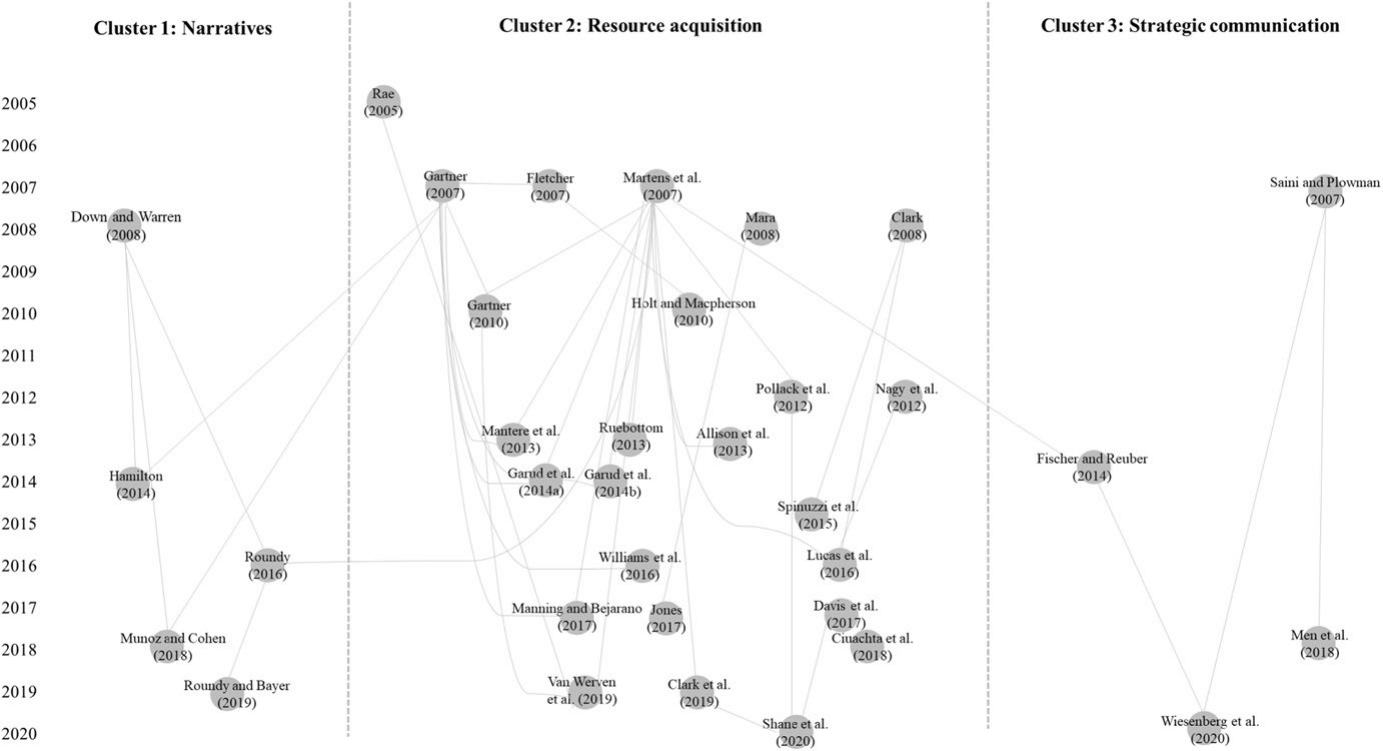
Historiograph of entrepreneurial communication research

Although our dataset covered nearly 50 years of publication, the intellectual structures of the field were still nascent. Figure 2 has already shown that, from 2004 onwards, there was continuous progress in the development of entrepreneurial communication research. This result was also reflected in our historiograph since coherent structures were clearly visible from 2004. From 1973 to 2005, the studies showed few interrelationships, so we focused on 2005 onward in this analysis. The results of our historiographic mapping are illustrated in Fig. 4 and show three core clusters overall.

Cluster 1, on the left side of the figure, focuses on narrative as a component of entrepreneurial communication for founders and other stakeholders (Down and Warren 2008; Hamilton 2014; Harmeling 2011; Muñoz and Cohen 2018; Roundy 2016; Roundy and Bayer 2019). In detail, this research cluster examined identity-building narratives (Down and Warren 2008) in entrepreneurial ecosystems (Roundy 2016; Roundy and Bayer 2019) or sustainable startups (Muñoz and Cohen 2018). Furthermore, this cluster also focused on the communication used by stakeholders when talking about failed startups (Mantere et al. 2013). Roundy’s (2016) and Gartner’s (2007) studies built a bridge between the narrative cluster and the second cluster, initialized by Martens et al. (2007).

Cluster 2, in the middle of the figure, primarily considered communication dedicated to acquiring resources and built on the foundation of earlier work by Martens et al. (2007), which formed the indirect or direct cornerstone of this cluster (Allison et al. 2013; Lucas et al. 2016; Manning et al. 2020; Manning and Bejarano 2017; Pollack et al. 2012; Shane et al. 2020; van Werven et al. 2019). In this regard, communication was often considered in the context of an investor pitch to examine the communication skills of entrepreneurs (Clark 2008), their rhetoric strategies (Holt and Macpherson 2010; van Werven et al. 2019), the communication of passion (Davis et al. 2017; Lucas et al. 2016; Shane et al. 2020), specific behavioral factors (e.g., preparedness; Pollack et al. 2012), product creativity (Davis et al. 2017), usage of storytelling (Manning et al. 2020), pitch deck design (Spinuzzi et al. 2015), or figurative language (Clarke et al. 2019). Furthermore, marketing communication (Mara 2008), entrepreneurial coachability (Ciuchta et al. 2018), cultural empowerment (Jones 2017), and impression management (Nagy et al. 2012) are part of this cluster. We also noted that this cluster included entrepreneurial learning (Rae 2005) as well as considering the narratives used in communication to convey entrepreneurial stories (Fletcher 2007) and entrepreneurial intentions (Gartner 2010).

Cluster 3, on the right of the figure, was also initiated by Martens et al. (2007) but connected to Fischer and Reuber (2014) and their study of online entrepreneurial communication. It also built a bridge between entrepreneurial and strategic communications. Although the core of previous research considered entrepreneurship and associated communication, the Wiesenberg et al. (2020) paper opened this up from a strategic communication perspective. Saini and Plowman (2007) initiated another trend in this cluster with their study on internal communication, which forged a link between strategic communication (Wiesenberg et al. 2020) and further studies on entrepreneurial leadership communication (Men et al. 2018).

This historiographic mapping showed that a key focus was entrepreneurial communication and its connection to resource acquisition, especially in investor relationships. Furthermore, this cluster was linked to the developing research stream of internal communication with startup employees and the overarching conceptualization of entrepreneurial strategic communication. Moreover, the results showed that entrepreneurial communication research was strongly related to the study of narratives used by entrepreneurs and to describe entrepreneurs (e.g., stakeholders from the entrepreneurial ecosystem). This also expanded the understanding of entrepreneurial communication, including communication about entrepreneurs and entrepreneurship.

In summary, this analysis reflects the results of the previous analysis. First, entrepreneurial communication is a young field whose structures are just emerging. Second, communication with stakeholders is a central focus of the research, especially in generating resources for startups.

## Discussion and agenda for future entrepreneurial communication research

### Discussion of the main findings

Our study used descriptive and bibliometric analyses with science mapping to structure previous entrepreneurial communication research. The descriptive results highlighted that entrepreneurial communication is on the upswing, with a growing number of publications. Although the term entrepreneurial communication suggests that both entrepreneurship and communication research are integrated, the latter has only recently been introduced, as the results for the top contributing journals showed. For example, a significant increase in 2022 was associated with the *International Journal of Strategic Communication*. We also observed a strong influence of researchers with a communication focus among the essential authors according to the number of articles.

Moreover, this view was further reinforced by the historiograph, which also showed a communication perspective increase, especially after 2020 (Men et al. 2021a; Wiesenberg et al. 2020). Accordingly, entrepreneurial communication has increasingly developed into a cross-disciplinary topic similar to that seen, for example, in entrepreneurial marketing research (Hills et al. 2008; Most et al. 2018). This has resulted in theoretical concepts from both disciplines being integrated into and developing this research stream.

Although various dimensions of entrepreneurial communication have already been studied, the conceptual understanding is still ambiguous (Gossel 2022). Against this background, we suggest the following definition that summarizes our findings and explains the conceptual roots of entrepreneurial communication.

#### **Definition**

Entrepreneurial communication involves all information-sharing efforts by entrepreneurs with key stakeholder groups such as investors, employees, customers, and the larger public to help successfully establish and grow the startup.

This definition includes essential areas of previous conceptual understanding (see Gossel 2022 for a recent conceptual review). First, many organizational tasks are directly handled by entrepreneurs in the early stages of their startups and concern the means they use to communicate with different stakeholders and address different topics and information needs in light of their target audiences; for example, their presentations to investors in the context of startup pitches (Clark 2008), the hiring and management of startup employees (Men et al. 2021a), or internal startup communications (Wolf et al. 2022). Hence, our definition includes investor relations and investor communication (Moritz et al. 2015), employee relations and leadership communication (Men et al. 2021b), and public relations for communicating with the community (Chen et al. 2021). Second, building on this with a focus on entrepreneurs, our definition addresses the associated skills, as communication is considered a vital entrepreneurial skill (Gossel 2022; Hill and Levenhagen 1995; Martin 2009). Third, from an overarching point of view, entrepreneurial communication includes a strategic perspective (Godulla and Men 2022; Rudeloff et al. 2022; Wiesenberg et al. 2020). Thus, overall, communication contributes to the survival of an organization and ensures its continued existence (Zerfass et al. 2018), meaning that, according to our definition, entrepreneurial communication plays a vital role in keeping startups running and growing. In summary, entrepreneurial communication is the overarching term used to describe how entrepreneurs communicate with different audiences in different contexts to ensure the operation of their startups. Nevertheless, the term entrepreneurial communication could also be defined in a broader sense, as our results revealed that it generally refers to communication in a startup ecosystem and, thus, to both the communication used by entrepreneurs themselves and the communication that takes place via entrepreneurs (i.e., the stories and narratives that are created by and used to describe entrepreneurs).

The themes identified in our descriptive analyses and the findings from our science mapping showed that entrepreneurial communication had produced several core research streams. First, entrepreneurial communication related to resource acquisition to obtain both financial (Parhankangas and Ehrlich 2014) and human resources (Men et al. 2021a) was a motor theme for the development of the field. In this context, it proved vital for establishing and maintaining relationships with stakeholders, with a particular focus on acquisition. These findings are consistent with the literature review conducted by Wiesenberg et al. (2020), who also identified this cluster. Second, another cluster dealt primarily with online communication, especially through social media channels (Chen et al. 2021; Fischer and Reuber 2014). This cluster label differed from previous results in that Wiesenberg et al. (2020) clustered such communication together with external communication. Third, the influence of communication researchers and journals has made strategic communication the main focus of recent research (Gossel 2022; Rudeloff et al. 2022; Wiesenberg et al. 2020). Again, we observed links to Wiesenberg et al.'s (2020) study, but this path has become much more comprehensive through further new studies. Fourth, on a conceptual level, we found that entrepreneurial communication is driven by narratives (Gartner 2007; Mantere et al. 2013; Roundy and Bayer 2019), which have been examined to explore the communication between entrepreneurs and their stakeholders. In this context, we also noted that entrepreneurial communication did not exclusively refer to the communication of founders; in a broader sense, it also included narratives by and about entrepreneurs.

### Developing an integrative framework of entrepreneurial communication

**Fig. 5:**
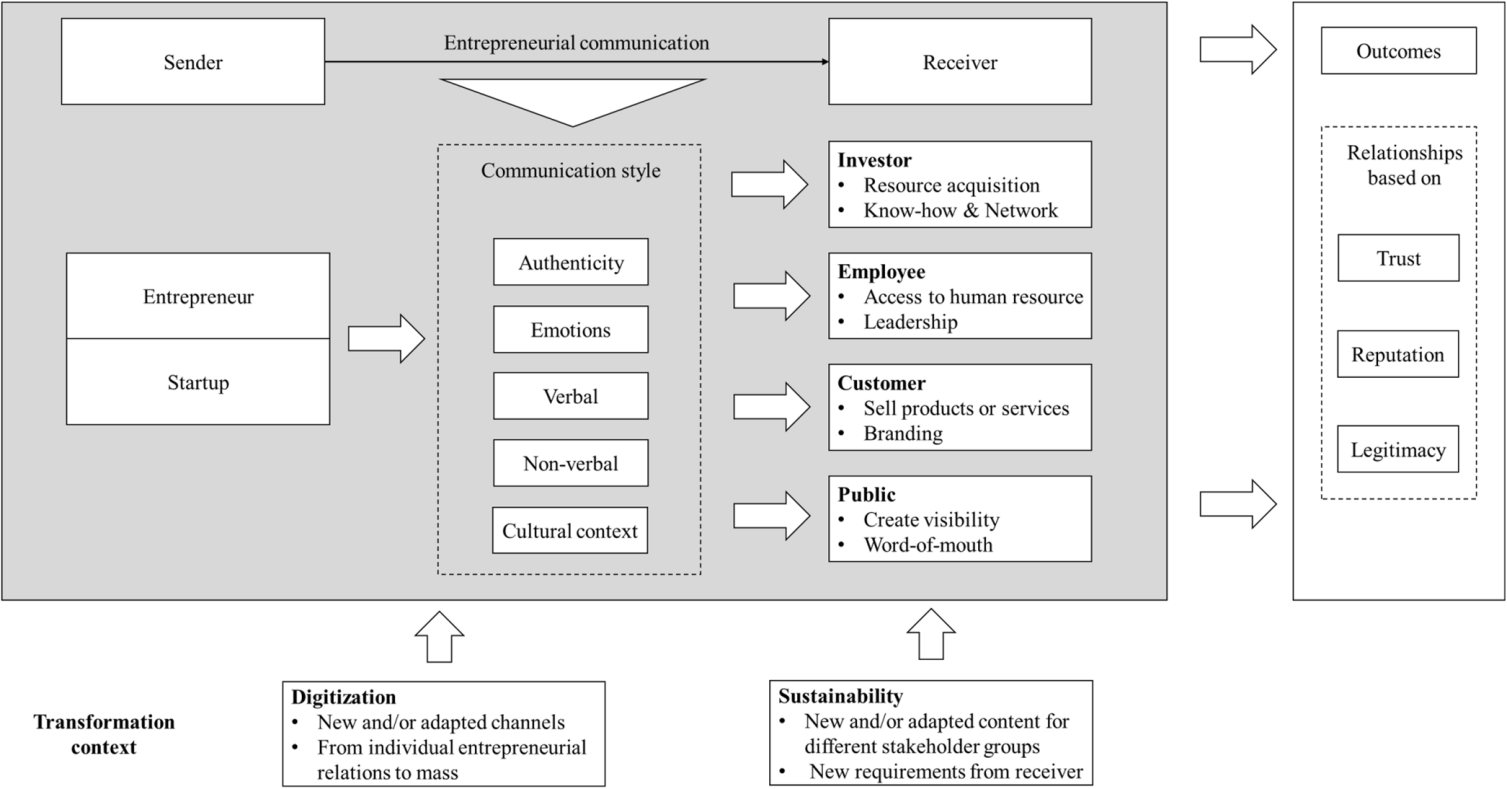
Framework of entrepreneurial communication research

Based on the findings of this bibliometric analysis, we developed an integrative framework in Fig. 5 to summarize the structure of entrepreneurial communication research, create an overarching picture of the current knowledge and show our understanding. The structure of this framework is described below.

In synthesizing the findings, we build our framework on the classic sender-receiver model (sometimes known as Shannon–Weaver-model; see also Shannon (1948)). At its core, this model provides the information source on the one side: the message's sender. Furthermore, on the other side of this model, the submitted message arrives at the receiver.

The left side of our model shows the sender, the entrepreneur and/or the startup in entrepreneurial communication. Previous studies show that communication is examined chiefly from the perspective of entrepreneurs (Balachandra et al. 2021; Clark 2008; Clingingsmith et al. 2022; Men et al. 2021a). Furthermore, the startup as an organization is also a sender of messages (Chen et al. 2021; Fischer and Reuber 2014). In addition, we have found that factors affect the preparation of information as communication style. On the one hand, these include authenticity (Men 2021) and emotion (Fernández-Vázquez and Álvarez-Delgado 2020). Authenticity appeals to openness and transparency and is essential for startup employees, for example, to create trusting relationships (Men et al. 2018). Furthermore, the sender's expressed emotions are vital to the receiver (Scherer 2003). In the investor pitch, for example, they support entrepreneurs' arguments and show a positive reaction from the receiver (Fernández-Vázquez and Álvarez-Delgado 2020).

On the other hand, previous studies show that entrepreneurial communication occurs verbally through spoken words (van Werven et al. 2019) and additionally through non-verbal signals (Clarke et al. 2019). Moreover, previous studies show that entrepreneurial communication occurs in different cultural settings (e.g., China, Morocco, and Germany). The sender's messages about his style then reach the receiver in the entrepreneurial communication framework. As mentioned in the previous sections, the investors (Kollmann and Kuckertz 2006) and employees (Men et al. 2021a) are two vital target groups and receivers of the messages. With investor communication, entrepreneurs address their need for financial resources (Martens et al. 2007) supplemented by investors' non-financial capital. Employee communication is also relevant for resources, in this case, for human resources (Wiesenberg et al. 2020). Moreover, as entrepreneurs act as leaders, leadership communication is another part of entrepreneurial communication (Men et al. 2021b). Furthermore, previous research shows that entrepreneurial marketing communication is used to address customers as another group of receivers (Wiesenberg et al. 2020) and to build the startup brand (Chaudhri et al. 2022). The fourth receiver is the general public for public relations activities (Chen et al. 2021; Gray et al. 2004) to show presence against this audience and influence their word-of-mouth through dialog communication (Chen et al. 2021).

Current developments in digitization and sustainability have highlighted transformation-driven communication as an external context of entrepreneurial communication that influences the sender-receiver relationship. Digital transformation has provided new ways of sharing information online and thus supports entrepreneurs' digital communication with their stakeholders (Fischer and Reuber 2014; Meurer et al. 2022), which leads to the emergence of new channels through which to communicate. In the context of sustainability, a complementary stream of research is developing that considers how startups take this into account (Constantin and Kavoura 2022; Simon and Ettl 2019). In this process, the content of communication changes.

 The communication of sender and receiver leads especially to stakeholder relationships. Previous publications note entrepreneurs' need to build trust (Welter 2012), reputation (Rode and Vallaster 2005) as well as legitimacy with their stakeholders (Nagy et al. 2012). Entrepreneurial communication addresses these challenges as our framework shows on the right side with the outcomes and leads to relationships based on these vital concepts; trust (Kaiser and Berger 2021; Lakeman et al. 2021), reputation (Abeysekera 2019) and legitimacy (Huang-Horowitz and Evans 2020; Nagy et al. 2012). Enabling these relationships leads to the initial argument that entrepreneurial communication plays a central role in any startup's survival (see Zerfass et al. 2018 for the relevance of communication for organizations).

### Development of a future research agenda

The results of our study showed that the research stream of entrepreneurial communication is still in its growth stage, and many unanswered questions remain (Godulla and Men 2022; Wiesenberg et al. 2020). Consequently, our analyses highlight research paths that have not yet been taken (Block and Fisch 2020), and the study can serve to formulate a future research agenda and stimulate future entrepreneurial communication research. Our recommendations are based on the framework shown in Fig. 5 to elucidate how the identified structures can be used to develop the field further.

#### Theoretical development of the field

Across the 383 identified studies, the focus of entrepreneurial communication research was on entrepreneurs' communication, expanded to include the communication of other actors in the overall entrepreneurial ecosystem. Therefore, previous research has focused on how entrepreneurs communicate and employ various sub concepts of communication. This opens up the question of the extent to which entrepreneurial communication is related exclusively to the entrepreneurial ecosystem. In our discussion, we have already indicated that structures resembling those of entrepreneurial marketing research are developing. Research on entrepreneurial marketing has discussed their conceptual roots in the context of entrepreneurs (Gruber 2003) and in a broader context as an innovative concept that can be used independently of entrepreneurship (e.g., for established organizations) (Morris and Paul 1987). In this vein, Kraus et al. (2010) referred to entrepreneurial marketing as “marketing activities with an entrepreneurial mindset” (p. 2). This view could also apply to entrepreneurial communication, and although this research stream has examined communication in established organizations, it has primarily considered innovative cases demonstrating an entrepreneurial mindset. Against this backdrop, Gossel (2022) pointed out that a view focused solely on startups may not be sufficient. We found that a broad conceptual understanding of entrepreneurial communication also captured stakeholders' communication about entrepreneurs using entrepreneurial stories. In addition, future research should clarify the conceptual relationships and differences between entrepreneurial marketing and communication. Thus far, we do not understand well how these two concepts are linked or where researchers see differences and similarities.

Based on recent developments in this research stream, there are indications that the influence of communication research is increasing. This means, for future research, that further concepts from communication can be integrated, leading to the following research paths:

- A better understanding of the role of the entrepreneurial mindset in the communication of established organizations.
- Clarification of the relationship between entrepreneurial communication and entrepreneurial marketing.
- A deeper understanding of the communication discipline and its influence on entrepreneurial communication.

#### Investor communication

Previous research has shown that, as a critical task, entrepreneurs use communication to build investor relationships (Wiesenberg et al. 2020). However, research on investor communication has focused mainly on the pre-investment phase of the relationship and analyzed communication in pitch presentations—an initial task necessary to convince investors of their support (van Werven et al. 2019). This is surprising, as we know from the literature on startup financing that entrepreneur–investor relationships have different phases (Maxwell et al. 2011; Tyebjee and Bruno 1984). Against this background, it is therefore essential for research to include the post-investment phase of investor communication. By analyzing post-investment communication, studies on this phase would help entrepreneurs to take a holistic view of investor communication.

Furthermore, the research on individual actors is very scattered: communication with venture capitalists (Kollmann and Kuckertz 2006), with business angels (Parhankangas and Ehrlich 2014), or online with crowdfunding investors (Moritz et al. 2015). Furthermore, general stakeholders are also involved, which is why there are opportunities for further investigation of investor communication (Fischer and Reuber 2014). The few studies that dealt explicitly with investor communication were distributed across a small number of investor types, but this research could be extended by the fact that investor types are in a state of flux, and the investigation of previously unexplored investors could enrich the field (Bellavitis et al. 2017) Forms of financing could include accelerators (Crișan et al. 2021), family offices (Zellweger and Kammerlander 2015), or initial coin offerings (Fisch 2019). Entrepreneurs must also communicate with investors regarding these financing forms, so there are opportunities for practice and research to investigate this heterogeneity in investor communication. Future studies could include individual investors, the structures of startups' general investor communication strategies, and how different types of investors influence them.

Our study observed an increasing trend in online entrepreneurial communication through social media, implying that these channels also play a relevant role in communication (Fischer and Reuber 2014; Olanrewaju et al. 2020). In the context of investor communications, however, this area has so far been underexplored, so we know less about using social media for this purpose. This raises the question of what role social media play in investor communications for startups and what requirements individual investors (e.g., venture capitalists, business angels, family offices, accelerators, and initial coin-offering investors) have of them. Overall, these considerations offer the following research paths for future entrepreneurial communication research in the context of investors:

- Clarification of communication structures in the post-investment phase of entrepreneur–investor relationships.
- Expansion of entrepreneurial investor communication to newer forms of financing (e.g., accelerators, family offices, or initial coin offerings).
- Inclusion of the digital context of social media channels in analyses of entrepreneurial communication in investor relations.

#### Employee and leadership communication

We have also mentioned other target groups besides investors. While future research on entrepreneurial investor communication could continue to focus on external stakeholders, entrepreneurs also need to provide internal stakeholders with information and address them in their communication (Wiesenberg et al. 2020). In this context, Godulla and Men (2022) mentioned entrepreneurial leadership communication as a relevant field of research. One possibility is for future studies to consider the aftermath of the COVID-19 pandemic (see for example Kuckertz and Brändle 2022 for an recent review). This pandemic has led to uncertainty among employees in organizations, especially since March 2020 (the pandemic declaration), regarding what will happen in the future. It has also changed the leadership context from personal leadership to digital leadership. Although we mentioned an increase in publications after 2020 (the pandemic outbreak) in Sect. 3.1, we identified no stronger content link to crisis research. Therefore, entrepreneurial crisis management focusing on communication is not very visible in previous research and is still largely unexplored (Kaiser and Kuckertz 2023), which is relevant not only for the COVID-19 context but for crisis contexts in general. Similar to investors, this raises the question of how social media is integrated into employee communication. Furthermore, communication is closely linked with other concepts, such as trust, which opens up further opportunities for future research to investigate how entrepreneurial leadership communication needs to be designed to foster employee trust. These research paths could include the following considerations of entrepreneurial leadership communication:

- A better understanding of crises (e.g., the COVID-19 pandemic, natural disasters, or financial crises) and “black swan” events to explore their influences on entrepreneurial communication.
- Clarification of the role of social media channels in entrepreneurial communication with employees.
- A better understanding of other relationship concepts (e.g., trust) and their connections to entrepreneurial communication.

#### Technological impact on entrepreneurial communication

Communication is influenced by the context and environment in which it occurs. This analysis illustrated that technological change is changing communication, whether for online communication via social media (Fischer and Reuber 2014; Pakura et al. 2020) or online financing via crowdfunding (Moritz et al. 2015), to give two examples. However, according to current developments, this seems to be only the beginning because other technologies are starting to influence communication. Thus, in line with Godulla and Men (2022), new technologies are opening up new research streams. One example is artificial intelligence (AI), which involves machine learning and neural networks. On the one hand, early AI-based models make it possible for texts to be written by this technology (Short and Short 2023). Also, researchers have recently shown that technology can generate images from text (Saharia et al. 2022), thus transforming visual communication. On the other hand, AI is changing the possibilities for analyzing communication by, for example, enabling texts to be analyzed for their content (Antretter et al. 2019). However, thus far, entrepreneurial communication research has discovered little about the use of AI from the perspective of entrepreneurs and stakeholders, such as investors (Short and Short 2023). Therefore, the question of the AI status quo in entrepreneurial communication strategies arises. Furthermore, few studies have been conducted on how much stakeholders (e.g., investors) use this technology for communication analysis. In summary, based on these considerations, the following research paths can be explored:

- Exploration of the possible applications of AI in the context of entrepreneurial communication.
- A better understanding of AI and its influence on entrepreneurial communication strategies.
- Consideration of the perspectives of entrepreneurs' stakeholders (e.g., investors or employees) and their possible usage of AI relative to entrepreneurs' communication.

#### Entrepreneurial communication in the context of sustainability

Although digital transformation is often a central field of research (see our analyses and future research agenda), sustainability transformation is also a critical field of action. A research stream on sustainable entrepreneurship has developed (Berger and Blanka 2023; Hinderer and Kuckertz 2022; Ruebottom 2013) that considers, for example, social innovations, sustainable business models, or impact investing. Thus far, the extent to which these developments affect entrepreneurial communication research has been largely unexplored. This is surprising, as there are interesting and relevant research questions that could advance the field. For example, previous studies have shown that stakeholders pay attention to sustainability (Lortie et al. 2022). In entrepreneurial finance, a separate area of impact investing has been developed that encourages these investors to make sustainable investments (Block et al. 2021). This leads to the question of the extent to which these financial stakeholders also place different demands on entrepreneurial communication inseparably from the context of communication content. However, this question applies equally to employees. These potential research questions could also be explored with entrepreneurs regarding sustainable startups. Previous studies have shown that different groups of people communicate differently (Obschonka et al. 2017). So far, little is known about the extent to which entrepreneurs with sustainable startups exhibit different characteristics when communicating. Building on these considerations, we derived the following research opportunities:

- A better understanding of stakeholders' sustainable requirements for entrepreneurial communication.
- Exploration of sustainable entrepreneurial communication and how this differs from traditional approaches to entrepreneurial communication.
- Understanding the communication of sustainable entrepreneurs and how it differs from entrepreneurs using other business models.

## Practical implications

Beyond the development of a future research agenda, our study's findings also provide implications for practice. First, entrepreneurial communication is identified as an essential tool for building relationships. Therefore, entrepreneurs should know that communication is necessary and connected with other vital concepts such as trust and legitimacy. Communication should therefore be used purposefully for visibility and to gain the trust of others, reputation, and achieve legitimacy of their business models. In this vein, our model gives an overview of essential factors of the communication style. Even though time is often a critical factor in startups and many tasks depend on the entrepreneurs, communication should be prioritized to build relationships. Second, our results show that different target groups are addressed with entrepreneurial communication. Entrepreneurs should remember this in their communication and be clear about whom they are addressing (e.g., customer, employee, investor) and what the target group expects. In this vein, entrepreneurs should create communication plans and concepts for each audience to address them in the right place and with the right message—target-group-specific communication. Third, communication is a skill and task often connected with the entrepreneur and their role as a startup leader. However, not every entrepreneur has the same skill set, so permanent learning plays a vital role in entrepreneurial communication. Our study shows that new requirements of society and economies (e.g., sustainability, digitization) influence communication, so communication skills must be permanently examined and developed further if necessary (e.g., with upskilling). In practice, entrepreneurial communication must, therefore, constantly adapt to changes and consider these requirements (e.g., content, medium).

## Limitations

Our study has some limitations, which are explained in more detail below. First, although we used Scopus—a relevant database that has been a valuable tool for many previous entrepreneurship studies—this is not the only database that could have been used. Therefore, this study considered only articles included in Scopus or identifiable through it. Second, when selecting the search terms, we included many aspects of entrepreneurial communication in the keywords. However, other researchers could have written about entrepreneurial communication but chosen other terms for their article titles. Third, we limited our bibliometric analysis to peer-reviewed articles; thus, this sample did not include articles published in gray literature. Fourth, this study was limited to bibliometric analysis. We did not conduct a detailed content analysis for a structured literature review. Fifth, similar to previous bibliometric analyses (Block et al. 2020), we did not use a full year as the end date but instead conducted the analysis in October, so the analysis and interpretation of citations must take this into account.

## Conclusion

With this bibliometric analysis, a further step has been taken to systematically capture the research field of entrepreneurial communication (Godulla and Men 2022). Although Wiesenberg et al. (2020) provided an overview of the six dimensions of communication in their structured literature review, we extended this with a bibliometric approach to show the structure of entrepreneurial communication research. Thus, this paper provides an orientation for future research in entrepreneurial communication.

## Figures and Tables

**Fig. 6:**
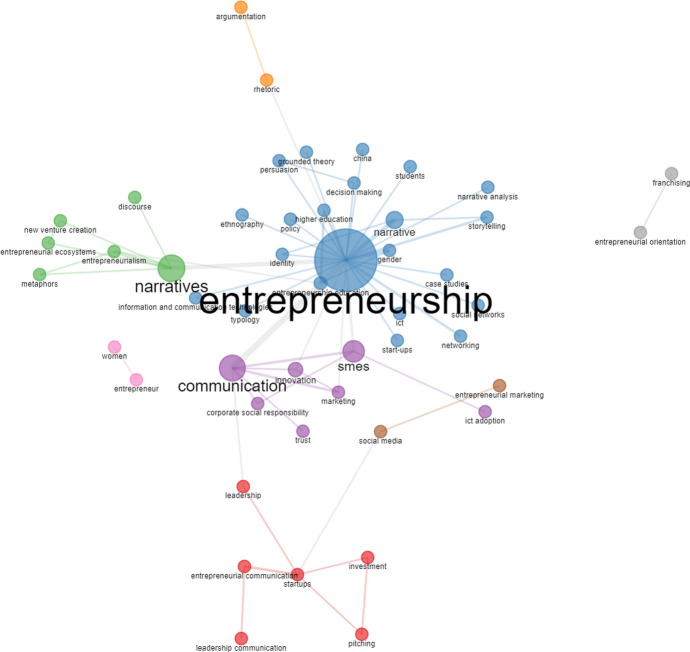
Co-occurrence analysis with keywords

## Appendix

See Fig. 6.

## Footnotes

1 We are aware that it is common in some bibliometric analyses to include only complete years in the analysis. Thus, if the search query takes place in the middle of the year, as in our study, the search year is often excluded for further analysis. However, since this research stream is still developing, we did not want to exclude any relevant articles by using an artificial cut-off. In particular, in a first initial screening, we discovered that an important special issue on entrepreneurial communication was published in spring 2022, which would be excluded with a year cut-off. We therefore followed the study by Block et al. (2020), who conducted a bibliometric analysis including March 2019.

2 Figure 6 in the Appendix shows the traditional co-occurrence network for our sample as an extension of this analysis.

## References

[CR1] Abeysekera I (2019). How best to communicate intangible resources on websites to inform corporate-growth reputation of small entrepreneurial businesses. J Small Bus Manag.

[CR2] Allison TH, McKenny AF, Short JC (2013). The effect of entrepreneurial rhetoric on microlending investment: an examination of the warm-glow effect. J Bus Ventur.

[CR3] Alnajem M, Mostafa MM, ElMelegy AR (2021). Mapping the first decade of circular economy research: a bibliometric network analysis. J Ind Prod Eng.

[CR4] Anand A, Argade P, Barkemeyer R, Salignac F (2021). Trends and patterns in sustainable entrepreneurship research: a bibliometric review and research agenda. J Bus Ventur.

[CR5] Antretter T, Blohm I, Grichnik D, Wincent J (2019). Predicting new venture survival: a Twitter-based machine learning approach to measuring online legitimacy. J Bus Ventur Insights.

[CR6] Aria M, Cuccurullo C (2017). Bibliometrix: an R-tool for comprehensive science mapping analysis. J Informetr.

[CR7] Aria M, Cuccurullo C, D’Aniello L, Misuraca M, Spano M (2022). Thematic analysis as a new culturomic tool: the social media coverage on COVID-19 pandemic in Italy. Sustainability.

[CR8] Balachandra L, Fischer K, Brush C (2021). Do (women's) words matter? The influence of gendered language in entrepreneurial pitching. J Bus Ventur Insights.

[CR9] Bellavitis C, Filatotchev I, Kamuriwo DS, Vanacker T (2017). Entrepreneurial finance: new frontiers of research and practice. Venture Cap.

[CR10] Berger ESC, Blanka C (2023). Comprehensive and multifaceted perspectives on sustainability, urban studies, and entrepreneurship. Small Bus Econ.

[CR11] Block JH, Fisch C (2020). Eight tips and questions for your bibliographic study in business and management research. Manag Rev Q.

[CR12] Block J, Fisch C, Rehan F (2020). Religion and entrepreneurship: a map of the field and a bibliometric analysis. Manag Rev Q.

[CR13] Block JH, Hirschmann M, Fisch C (2021). Which criteria matter when impact investors screen social enterprises?. J Corp Finance.

[CR14] Bretas VP, Alon I (2021). Franchising research on emerging markets: bibliometric and content analyses. J Bus Res.

[CR15] Budler M, Župič I, Trkman P (2021). The development of business model research: a bibliometric review. J Bus Res.

[CR16] Chapple D, Pollock N, D’Adderio L (2021). From pitching to briefing: extending entrepreneurial storytelling to new audiences. Organ Stud.

[CR17] Chaudhri V, Pridmore J, Mauck C (2022). Assembling the start-up brand: a process framework for understanding strategic communication challenges. Int J Strateg Commun.

[CR18] Chen ZF, Ji YG, Men LR (2021). Effective social media communication for startups in China: antecedents and outcomes of organization–public dialogic communication. New Media Soc.

[CR19] Ciuchta MP, Letwin C, Stevenson R, McMahon S, Huvaj MN (2018). Betting on the coachable entrepreneur: signaling and social exchange in entrepreneurial pitches. Entrep Theory Pract.

[CR20] Clark C (2008). The impact of entrepreneurs' oral ‘pitch’ presentation skills on business angels' initial screening investment decisions. Venture Cap.

[CR21] Clarke JS, Cornelissen JP, Healey MP (2019). Actions speak louder than words: How figurative language and gesturing in entrepreneurial pitches influences investment judgments. Acad Manag J.

[CR22] Clingingsmith D, Drover W, Shane S (2022). Examining the outcomes of entrepreneur pitch training: an exploratory field study. Small Bus Econ.

[CR23] Cobo MJ, López-Herrera AG, Herrera-Viedma E, Herrera F (2011). An approach for detecting, quantifying, and visualizing the evolution of a research field: a practical application to the fuzzy sets theory field. J Informetr.

[CR24] Collewaert V, Vanacker T, Anseel F, Bourgois D (2021). The sandwich game: founder-CEOs and forecasting as impression management. J Bus Ventur.

[CR25] Constantin F, Kavoura A (2022). Digital entrepreneurship via sustainable online communication of dentistry profession, Oradea, Romania: a longitudinal analysis. Sustainability.

[CR26] Crișan EL, Salanță II, Beleiu IN, Bordean ON, Bunduchi R (2021). A systematic literature review on accelerators. J Technol Transf.

[CR27] Davis BC, Hmieleski KM, Webb JW, Coombs JE (2017). Funders' positive affective reactions to entrepreneurs' crowdfunding pitches: the influence of perceived product creativity and entrepreneurial passion. J Bus Ventur.

[CR28] Deyanova K, Brehmer N, Lapidus A, Tiberius V, Walsh S (2022). Hatching start-ups for sustainable growth: a bibliometric review on business incubators. Rev Manag Sci.

[CR29] Dolhey S (2019). A bibliometric analysis of research on entrepreneurial intentions from 2000 to 2018. J Res Mark Entrep.

[CR30] Donthu N, Kumar S, Mukherjee D, Pandey N, Lim WM (2021). How to conduct a bibliometric analysis: an overview and guidelines. J Bus Res.

[CR31] Down S, Warren L (2008). Constructing narratives of enterprise: clichés and entrepreneurial self-identity. Int J Entrep Behav Res.

[CR32] Fernández-Vázquez J-S, Álvarez-Delgado R-C (2020). The interaction between rational arguments and emotional appeals in the entrepreneurial pitch. Int J Entrep Behav Res.

[CR33] Fisch C (2019). Initial coin offerings (ICOs) to finance new ventures. J Bus Ventur.

[CR34] Fischer E, Reuber AR (2014). Online entrepreneurial communication: mitigating uncertainty and increasing differentiation via Twitter. J Bus Ventur.

[CR35] Fletcher D (2007). 'Toy story': the narrative world of entrepreneurship and the creation of interpretive communities. J Bus Ventur.

[CR36] Forliano C, de Bernardi P, Yahiaoui D (2021). Entrepreneurial universities: a bibliometric analysis within the business and management domains. Technol Forecast Soc Change.

[CR37] Garfield E (2004). Historiographic mapping of knowledge domains literature. J Inf Sci.

[CR38] Gartner WB (2007). Entrepreneurial narrative and a science of the imagination. J Bus Ventur.

[CR39] Gartner WB (2010). A new path to the waterfall: a narrative on a use of entrepreneurial narrative. Int Small Bus J.

[CR40] Garud R, Gehman J, Giuliani AP (2014). Contextualizing entrepreneurial innovation: a narrative perspective. Res Policy.

[CR41] Garud R, Schildt HA, Lant TK (2014). Entrepreneurial storytelling, future expectations, and the paradox of legitimacy. Organ Sci.

[CR42] Gassenheimer JB, Baucus DB, Baucus MS (1996). Cooperative arrangements among entrepreneurs: an analysis of opportunism and communication in franchise structures. J Bus Res.

[CR43] Ghura AS, Sharma GD, Pereira V, Islam N, Chopra R (2022). Corporate entrepreneurship champions: mapping the past and present states of the field for future advancements. Int J Entrep Behav Res.

[CR44] Godulla A, Men LR (2022). Start-up and entrepreneurial communication. Int J Strateg Commun.

[CR45] Gossel BM (2022). Analogies in entrepreneurial communication and strategic communication: definition, delimitation of research programs and future research. Int J Strateg Commun.

[CR46] Gray D, Davies F, Blanchard K (2004). Does use of public relations promote a higher growth rate in small firms?. Corp Commun.

[CR47] Gruber M (2003). Research on marketing in emerging firms: key issues and open questions. Int J Technol Manag.

[CR48] Hamilton E (2014). Entrepreneurial narrative identity and gender: a double epistemological shift. J Small Bus Manag.

[CR49] Harmeling SS (2011). Re-storying an entrepreneurial identity: education, experience and self-narrative. Educ Train.

[CR50] Hill RC, Levenhagen M (1995). Metaphors and mental models: sensemaking and sensegiving in innovative and entrepreneurial activities. J Manag.

[CR51] Hills GE, Hultman CM, Miles MP (2008). The evolution and development of entrepreneurial marketing. J Small Bus Manag.

[CR52] Hinderer S, Kuckertz A (2022). The bioeconomy transformation as an external enabler of sustainable entrepreneurship. Bus Strategy Environ.

[CR53] Hinderer S, Brändle L, Kuckertz A (2021). Transition to a sustainable bioeconomy. Sustainability.

[CR54] Hockerts K, Wüstenhagen R (2010). Greening Goliaths versus emerging Davids—theorizing about the role of incumbents and new entrants in sustainable entrepreneurship. J Bus Ventur.

[CR55] Holt R, Macpherson A (2010). Sensemaking, rhetoric and the socially competent entrepreneur. Int Small Bus J.

[CR56] Huang L, Knight AP (2017). Resources and relationships in entrepreneurship: an exchange theory of the development and effects of the entrepreneur–investor relationship. Acad Manag Rev.

[CR57] Huang-Horowitz NC, Evans SK (2020). Communicating organizational identity as part of the legitimation process: a case study of small firms in an emerging field. Int J Bus Commun.

[CR58] Johnson MP, Schaltegger S (2020). Entrepreneurship for sustainable development: a review and multilevel causal mechanism framework. Entrep Theory Pract.

[CR59] Jones NN (2017). Rhetorical narratives of black entrepreneurs: the business of race, agency, and cultural empowerment. J Bus Tech Commun.

[CR60] Kaiser M, Berger ESC (2021). Trust in the investor relationship marketing of startups: a systematic literature review and research agenda. Manag Rev Q.

[CR61] Kaiser M, Kuckertz A (2023) Emotional robustness in times of crisis: the effects of venture funding on the digital communication styles of entrepreneurs. J Small Bus Enterp Dev. 10.1108/JSBED-10-2022-0423 (**In Press**)

[CR01] Kalantari A, Kamsin A, Kamaruddin HS, Ale Ebrahim N, Gani A, Ebrahimi A, Shamshirband S (2017). A bibliometric approach to tracking big data research trends. J Big Data.

[CR62] Kollmann T, Kuckertz A (2006). Investor relations for start-ups: an analysis of venture capital investors' communicative needs. Int J Technol Manag.

[CR63] Kraus S, Harms R, Fink M (2010). Entrepreneurial marketing: moving beyond marketing in new ventures. Int J Entrep Innov Manag.

[CR02] Kraus S, Filser M, O’Dwyer M, Shaw E (2014) Social Entrepreneurship: An exploratory citation analysis. Rev Manag Sci 8:275–292. 10.1007/s11846-013-0104-6

[CR64] Kraus S, Mahto RV, Walsh ST (2021). The importance of literature reviews in small business and entrepreneurship research. J Small Bus Manag.

[CR65] Kuckertz A, Block J (2021). Reviewing systematic literature reviews: ten key questions and criteria for reviewers. Manag Rev Q.

[CR66] Kuckertz A, Brändle L (2022). Creative reconstruction: a structured literature review of the early empirical research on the COVID-19 crisis and entrepreneurship. Manag Rev Q.

[CR67] Lakeman FA, Walter N, Cleff T (2021). The impact of payment methods and payment-related marketing communications on e-commerce retailer trust—an empirical consumer analysis of Indonesian e-commerce start-ups. Int J Electron Bus.

[CR68] Lortie J, Cox KC, Roundy PT (2022). Social impact models, legitimacy perceptions, and consumer responses to social ventures. J Bus Res.

[CR69] Lucas K, Kerrick SA, Haugen J, Crider CJ (2016). Communicating entrepreneurial passion: personal passion vs. perceived passion in venture pitches. IEEE Trans Prof Commun.

[CR70] Manning S, Bejarano TA (2017). Convincing the crowd: entrepreneurial storytelling in crowdfunding campaigns. Strateg Organ.

[CR71] Manning P, Stokes P, Tarba SY, Rodgers P (2020). Entrepreneurial stories, narratives and reading—their role in building entrepreneurial being and behaviour. Int J Entrep Innov.

[CR72] Mantere S, Aula P, Schildt H, Vaara E (2013). Narrative attributions of entrepreneurial failure. J Bus Ventur.

[CR73] Mara A (2008). Ethos as market maker: the creative role of technical marketing communication in an aviation start-up. J Bus Tech Commun.

[CR74] Martens ML, Jennings JE, Jennings PD (2007). Do the stories they tell get them the money they need? The role of entrepreneurial narratives in resource acquisition. Acad Manag J.

[CR75] Martin DM (2009). The entrepreneurial marketing mix. Qual Mark Res.

[CR76] Maxwell AL, Jeffrey SA, Lévesque M (2011). Business angel early stage decision making. J Bus Ventur.

[CR77] Men LR (2021). The impact of startup CEO communication on employee relational and behavioral outcomes: responsiveness, assertiveness, and authenticity. Public Relat Rev.

[CR78] Men LR, Chen ZF, Ji YG (2018). Walking the talk: an exploratory examination of executive leadership communication at startups in China. J Public Relat Res.

[CR79] Men LR, Chen ZF, Ji YG (2021). Cultivating relationships with startup employees: the role of entrepreneurs’ leadership communication. Manage Commun Q.

[CR80] Men LR, Qin YS, Mitson R (2021). Engaging startup employees via charismatic leadership communication: the importance of communicating “vision, passion, and care”. Int J Bus Commu.

[CR03] Meurer MM, Waldkirch M, Schou PK, Bucher EL, Burmeister-Lamp K (2022) Digital affordances: howentrepreneurs access support in online communities during the COVID-19 pandemic. Small Bus Econ 58:637–663. 10.1007/s11187-021-00540-2 10.1007/s11187-021-00540-2 PMC8503734 38624988

[CR81] Moritz A, Block J (2022). Editorial to the special issue “structured literature reviews in entrepreneurship research-taking stock and setting the agenda”. Manag Rev Q.

[CR82] Moritz A, Block J, Lutz E (2015). Investor communication in equity-based crowdfunding: a qualitative-empirical study. Qual Res Financ Mark.

[CR83] Morris MH, Paul GW (1987). The relationship between entrepreneurship and marketing in established firms. J Bus Ventur.

[CR84] Most F, Conejo FJ, Cunningham LF (2018). Bridging past and present entrepreneurial marketing research. J Res Mark Entrep.

[CR85] Muñoz P, Cohen B (2018). Entrepreneurial narratives in sustainable venturing: beyond people, profit, and planet. J Small Bus Manag.

[CR86] Nagy BG, Pollack JM, Rutherford MW, Lohrke FT (2012). The influence of entrepreneurs' credentials and impression management behaviors on perceptions of new venture legitimacy. Entrep Theory Pract.

[CR87] Obschonka M, Fisch C, Boyd R (2017). Using digital footprints in entrepreneurship research: a Twitter-based personality analysis of superstar entrepreneurs and managers. J Bus Ventur Insights.

[CR88] Olanrewaju A-ST, Hossain MA, Whiteside N, Mercieca P (2020). Social media and entrepreneurship research: a literature review. Int J Inf Manag.

[CR89] Padilla AJ, Pagano M (1997). Endogenous communication among lenders and entrepreneurial incentives. Rev Financ Stud.

[CR90] Pakura S, Rudeloff C, Bekmeier-Feuerhahn S, Eggers F (2020). Communication management of start-ups: an empirical analysis of entrepreneurs' communication and networking success on Facebook. Int J Entrep Ventur.

[CR91] Parhankangas A, Ehrlich M (2014). How entrepreneurs seduce business angels: an impression management approach. J Bus Ventur.

[CR92] Park S-Y, Loo BT (2022). The use of crowdfunding and social media platforms in strategic start-up communication: a big-data analysis. Int J Strateg Commun.

[CR93] Pellegrini MM, Rialti R, Marzi G, Caputo A (2020). Sport entrepreneurship: a synthesis of existing literature and future perspectives. Int Entrep Manag J.

[CR94] Pollack JM, Rutherford MW, Nagy BG (2012). Preparedness and cognitive legitimacy as antecedents of new venture funding in televised business pitches. Entrep Theory Pract.

[CR95] Pollack JM, Barr S, Hanson S (2017). New venture creation as establishing stakeholder relationships: a trust-based perspective. J Bus Ventur Insights.

[CR96] Rae D (2005). Entrepreneurial learning: a narrative-based conceptual model. J Small Bus Enterp Dev.

[CR97] Ratten V (2020). Coronavirus and international business: an entrepreneurial ecosystem perspective. Thunderbird Int Bus Rev.

[CR98] Rode V, Vallaster C (2005). Corporate branding for start-ups: the crucial role of entrepreneurs. Corp Reput Rev.

[CR99] Roundy PT (2016). Start-up community narratives: the discursive construction of entrepreneurial ecosystems. HJ Entrep.

[CR100] Roundy PT, Bayer MA (2019). Entrepreneurial ecosystem narratives and the micro-foundations of regional entrepreneurship. Int J Entrep Innov.

[CR101] RStudio (2023) RStudio: integrated development environment for R. https://posit.co/products/open-source/rstudio/

[CR102] Rudeloff C, Bekmeier-Feuerhahn S, Sikkenga J, Barth A (2022). Conditions of one-way and two-way approaches in strategic start-up communication: a qualitative comparative analysis (QCA). Int J Strateg Commun.

[CR103] Ruebottom T (2013). The microstructures of rhetorical strategy in social entrepreneurship: building legitimacy through heroes and villains. J Bus Ventur.

[CR04] Saharia C, Chan W, Saxena S, Li L, Whang J, Denton E, Ghasemipour SKS, Ayan BK, Mahdavi SS, Lopes RG, Salimans T, Ho J, Fleet DJ, Norouzi M (2022) Photorealistic text-to-image diffusion models with deep language understanding. arXiv:2205.11487v1

[CR104] Saini S, Plowman K (2007). Effective communications in growing pre-ipo start-ups. J Promot Manag.

[CR105] Sapienza HJ, Korsgaard MA (1996). Procedural justice in entrepreneur–investor relations. Acad Manag J.

[CR106] Scherer K (2003). Vocal communication of emotion: a review of research paradigms. Speech Commun.

[CR107] Scopus (2022) Scopus-fact-sheet-2022_WEB

[CR108] Shane S, Drover W, Clingingsmith D, Cerf M (2020). Founder passion, neural engagement and informal investor interest in startup pitches: an fMRI study. J Bus Ventur.

[CR109] Shannon CE (1948). A mathematical theory of communication. Bell Syst Tech J.

[CR110] Short CE, Short JC (2023). The artificially intelligent entrepreneur: ChatGPT, prompt engineering, and entrepreneurial rhetoric creation. J Bus Ventur Insights.

[CR111] Simon J, Ettl K (2019). Entrepreneurs' views on corporate social responsibility communication in SMEs-insights from Germany. Int J Entrep Innov Manag.

[CR112] Sing J, Aust O (2022). Message machine: how communications will make you an unstoppable founder.

[CR113] Singh S, Walia N (2022). Momentum investing: a systematic literature review and bibliometric analysis. Manag Rev Q.

[CR114] Spinuzzi C, Nelson S, Thomson KS, Lorenzini F, French RA, Pogue G, Burback SD, Momberger J (2015). Remaking the pitch: reuse strategies in entrepreneurs' pitch decks. IEEE Trans Prof Commun.

[CR115] Statista (2020) Media usage during COVID-19 by country | Statista. https://www.statista.com/statistics/1106498/home-media-consumption-coronavirus-worldwide-by-country/. Accessed 21 April 2021

[CR116] Tyebjee TT, Bruno AV (1984). A model of venture capitalist investment activity. Manag Sci.

[CR117] van Eck NJ, Waltman L (2014). CitNetExplorer: a new software tool for analyzing and visualizing citation networks. J Informetr.

[CR118] van Werven R, Bouwmeester O, Cornelissen JP (2019). Pitching a business idea to investors: How new venture founders use micro-level rhetoric to achieve narrative plausibility and resonance. Int Small Bus J.

[CR119] Vogel B, Reichard RJ, Batistič S, Černe M (2021). A bibliometric review of the leadership development field: how we got here, where we are, and where we are headed. Leadersh Q.

[CR120] Wallnöfer M, Hacklin F (2013). The business model in entrepreneurial marketing: a communication perspective on business angels' opportunity interpretation. Ind Mark Manag.

[CR121] Welter F (2012). All you need is trust? A critical review of the trust and entrepreneurship literature. Int Small Bus J.

[CR122] Wiesenberg M, Godulla A, Tengler K, Noelle I-M, Kloss J, Klein N, Eeckhout D (2020). Key challenges in strategic start-up communication: a systematic literature review and an explorative study. J Commun Manag.

[CR123] Williams SD, Ammetller G, Rodriguez-Ardura I, Li X (2016). A narrative perspective on international entrepreneurship: comparing stories from the United States, Spain, and China. IEEE Trans Prof Commun.

[CR124] Wolf C, Godulla A, Beck L, Neubert LS (2022). The role of internal communication in start-ups: state of research and practical approaches. Int J Strateg Commun.

[CR125] Zellweger T, Kammerlander N (2015). Family, wealth, and governance: an agency account. Entrep Theory Pract.

[CR126] Zerfass A, Verčič D, Nothhaft H, Werder KP (2018). Strategic communication: defining the field and its contribution to research and practice. Int J Strateg Commun.

[CR127] Zupic I, Čater T (2015). Bibliometric methods in management and organization. Organ Res Methods.

